# Crystal structure of μ-carbonyl-1:2κ^2^ *C*:*C*-carbonyl-1κ*C*-(1η^5^-cyclo­penta­dien­yl)iodido-2κ*I*-[μ-2-(pyridin-2-yl)ethene-1,1-diyl-1κ*C* ^1^:2κ^2^ *N*,*C* ^1^]ironpalladium(*Fe*—*Pd*) benzene monosolvate

**DOI:** 10.1107/S2056989016019915

**Published:** 2017-01-01

**Authors:** Victor V. Verpekin, Arkadii Z. Kreindlin, Oleg V. Semeikin, Alexander F. Smol’yakov, Fedor M. Dolgushin, Oleg S. Chudin, Nikolai A. Ustynyuk

**Affiliations:** a Institute of Chemistry and Chemical Technology, Krasnoyarsk Research Center, Siberian Branch of the Russian Academy of Sciences, Akademgorodok 50-24, Krasnoyarsk, 660036, Russian Federation; b A. N. Nesmeyanov Institute of Organoelement Compounds, Russian Academy of Sciences, ul. Vavilova 28, Moscow 119991, Russian Federation

**Keywords:** crystal structure, μ-pyridyl­vinyl­idene, binuclear complex, iron, palladium

## Abstract

The reaction of Cp(CO)_2_FeI with 2-ethynyl­pyridine under Sonogashira conditions [5% PdCl_2_(PPh_3_)_2_, 10% CuI, THF–NEt_3_ (2:1)] afforded the title binuclear μ-pyridyl­vinyl­idene FePd complex (**FePd1**) as a benzene solvate, [FePd(C_5_H_5_)(C_7_H_5_N)I(CO)_2_]·C_6_H_6_, in a very low yield rather than the expected iron *o*-pyridyl­ethynyl complex Cp(CO)_2_Fe—C≡C-(2-C_5_H_4_N). The Fe and Pd atoms in **FePd1** are bridged by carbonyl and pyridyl­vinyl­idene ligands, the pyridyl N atom being bonded to the palladium atom. The use of equimolar amounts of PdCl_2_ increases the yield of **FePd1** to 12%. The reaction pathway leading to **FePd1** is proposed.

## Abstract 2

The title binuclear μ-pyridyl­vinyl­idene FePd complex (**FePd1**) was obtained from Cp(CO)_2_FeI and 2-ethynyl­pyridine in diisorpopyl­amine in the presence of PdCl_2_

## Chemical context   

Transition metal σ-pyridyl­ethynyl complexes attract considerable research inter­est since they can act as precursors for pyridyl­vinyl­idene complexes (Chou *et al.*, 2008 ▸) and as buildings blocks for supra­molecular assemblies in mol­ecular electronics (Le Stang *et al.*, 1999 ▸), as well as materials for non-linear optics (Wu *et al.*, 1997 ▸).
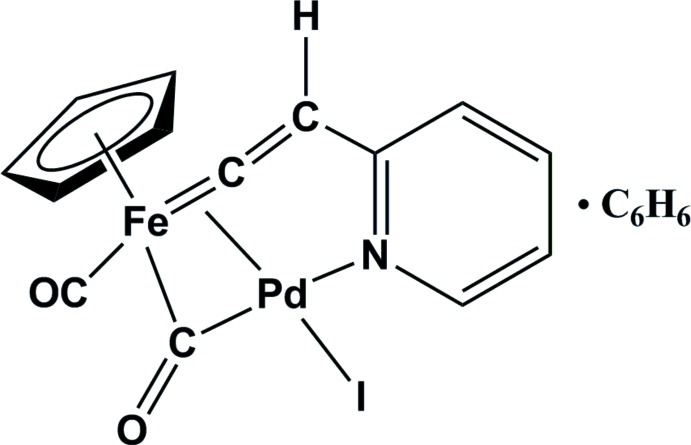


Since the presence of two Lewis base centres (C_β_ and N atoms) makes pyridyl­ethynyl complexes potential catalysts for electrochemical proton reduction (Valyaev *et al.*, 2007 ▸), we decided to study the CV behavior of the *o*-pyridyl­ethynyl iron complex Cp(CO)_2_Fe-C≡C-(2-C_5_H_4_N) in acidified solutions. The efficient preparation of iron aryl­ethynyls Cp(CO)_2_Fe-C≡C-Ar by Pd/Cu-catalyzed Sonogashira coupling of Cp(CO)_2_FeI (FpI) with terminal aryl­acetyl­enes HC≡C-Ar (Nakaya *et al.*, 2009 ▸) inspired us to study the reaction of Cp(CO)_2_FeI with *o*-pyridyl­acetyl­ene HC≡C-(2-C_5_H_4_N) under the same conditions (5% PdCl_2_(PPh_3_)_2_, 10% CuI, THF:NEt_3_ (2:1), 333 K). This reaction was found to afford no target complex. Instead, the binuclear FePd μ_2_-pyridyl­vinyl­idene complex (**FePd1**) was isolated in a yield of 2%. The yield increases to 12% using PdCl_2_ as an educt instead of (Ph_3_P)_2_PdCl_2_ and pure diisopropylamine as the solvent. The structure of **FePd1**, which crystallized as a benzene solvate [FePd(C_5_H_5_)(C_7_H_5_N)I(CO)_2_]·C_6_H_6_, was determined by X-ray diffraction.

**Figure 1:**
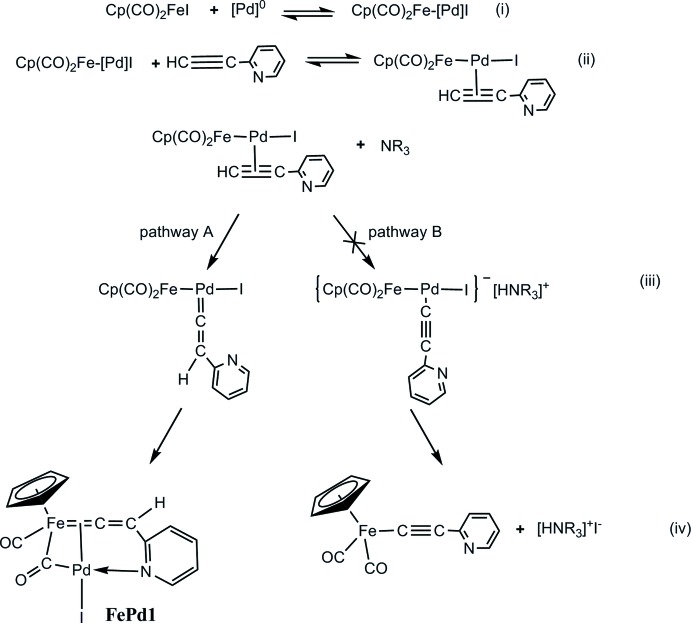
The reaction pathway.

Thus, while the alkynylation of FpI with terminal aryl­acetyl­ens HC≡C-Ar proceeds along the typical Sonogashira pathway to afford FpC≡C-Ar in reasonable yields (Nakaya *et al.*, 2009 ▸), the same reaction of *o*-pyridyl­acetyl­ene did not result in the Sonogashira alkynylation product, but afforded the binuclear complex **FePd1** where the metal atoms are bridged through the carbonyl and pyridyl­vinyl­idene ligands, the pyridyl nitro­gen atom being bound to the palladium atom. Although additional experimental and probably theoretical studies are needed to reveal the true reaction pathway, one can assume the formation of **FePd1** to be caused by the following successive steps in the palladium coordination sphere: (i) the oxidation addition of FpI at the Fe—I bond, (ii) the acetyl­ene–vinyl­idene rearrangement of the π-pyridyl­acetyl­ene ligand followed by (iii) insertion of the Cp(CO)_2_Fe-fragment into the Pd=C bond and accompanied by (iv) formation of the bridging carbonyl group and the Pd—N bond (Fig. 1 ▸, pathway A). Presumably, it is the Pd—N bond that efficiently stabilizes **FePd1**, thereby favoring pathway A. This stabilization cannot occur in the case of reactions of aryl­acetyl­enes, and the typical Sonogashira reaction proceeds *via* the formation of a pyridyl­ethynyl complex followed by the Fe—C-reductive elimination (Sonogashira, 1998 ▸) (Fig. 1 ▸, pathway B).

## Structural commentary   

**Figure 2:**
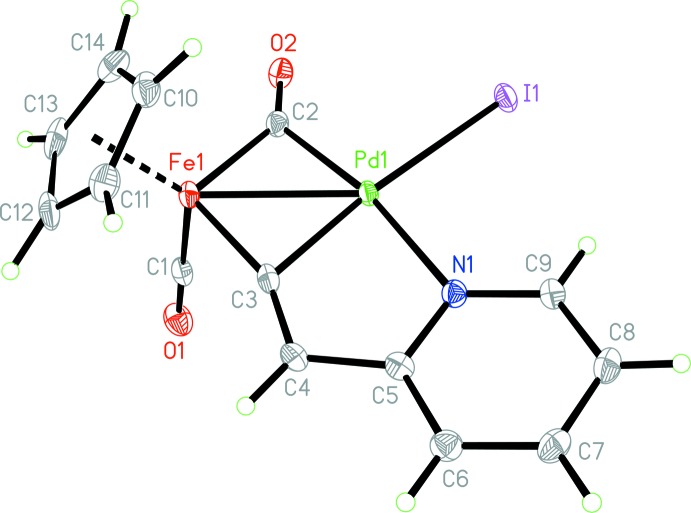
The mol­ecular structure of complex **FePd1** with the atom-numbering scheme. Displacement ellipsoids are drawn at the 50% probability level. The benzene solvent mol­ecule is omitted.

The mol­ecular structure of the title compound is shown in Fig. 2 ▸. The iron atom is coordinated by the cyclo­penta­dienyl ligand [the Fe—C distances lie between 2.075 (3) and 2.128 (3) Å and the Fe—Cp centroid distance is 1.731 (1) Å] and to two carbonyl ligands, one of which is terminal [the Fe1—C1—O1 angle is 177.6 (3)°] and the second one is bridging to the palladium atom [the Fe1—C2—O2 and O2—C2—Pd1 angles are 141.7 (2) and 137.0 (2)°, respectively, and the Fe1—C2 and Pd1—C2 distances are 1.942 (3) Å and 2.012 (3) Å, respectively]. In addition, the iron and palladium atoms are linked through the bridging pyridyl­vinyl­idene fragment coordinated by the C3 atom. The four-membered ring Fe1–C2–Pd1–C3 thereby formed is folded slightly by 11.61 (14)° along the Fe1⋯Pd1 line with a short metal–metal distance of 2.5779 (4) Å [for comparison the values of the covalent radii for these metals are *r*(Fe) = 1.32, *r*(Pd) = 1.39 Å; Cordero *et al.*, 2008 ▸]. The Fe1—C3 distance of 1.836 (3) Å is noticeably longer compared to the analogous distances in mononuclear iron vinyl­idene complexes: for example, 1.744 (4) Å in (η^5^-C_5_H_5_)Fe(SnPh_3_)(CO)(=C=CHPh) (Adams *et al.*, 1999 ▸) and 1.744 (9) Å in (η^5^-C_5_Me_5_)Fe(CO)(TMS)(=C=C(TMS)Ph) (Kalman *et al.*, 2014 ▸), and the Fe1—C3—C4 angle of 156.9 (2)° is noticeably deviated from linearity. At the same time, the Pd1—C3—C4 angle is 118.58 (19)°, which suggests an unsymmetrical coordination of the C3 atom to the iron and palladium atoms. This asymmetry can be explained by the η^2^-coordination of the Fe=C double bond to the palladium atom. It is noteworthy that in Fe–*M*-type binuclear μ_2_-vinyl­idene complexes, the coordination to the metal atoms is characterized by approximately equal values for the Fe—C—C and *M*—C—C angles [131.8–145.3° according to a CCDC (Groom *et al.*, 2016 ▸) search]. The C3—C4 distance of 1.328 (4) Å in the vinyl­idene fragment corres­ponds with typical C=C double-bond lengths in olefins. Besides coordination to C3, the palladium atom binds to the pyridyl­vinyl­idene fragment *via* the nitro­gen atom of the pyridine ring to a five-membered chelating ring (the ring is almost planar and the maximum deviation from the mean plane is 0.02 Å for atoms C3 and C4). The iodine atom completes the coordination sphere of the 16-electron palladium atom, which corresponds to a slightly distorted square-planar geometry [the dihedral angle between the N1/Pd1/C3 and I1/Pd/C2 planes is 3.2 (1)°].

## Supra­molecular features   

**Figure 3:**
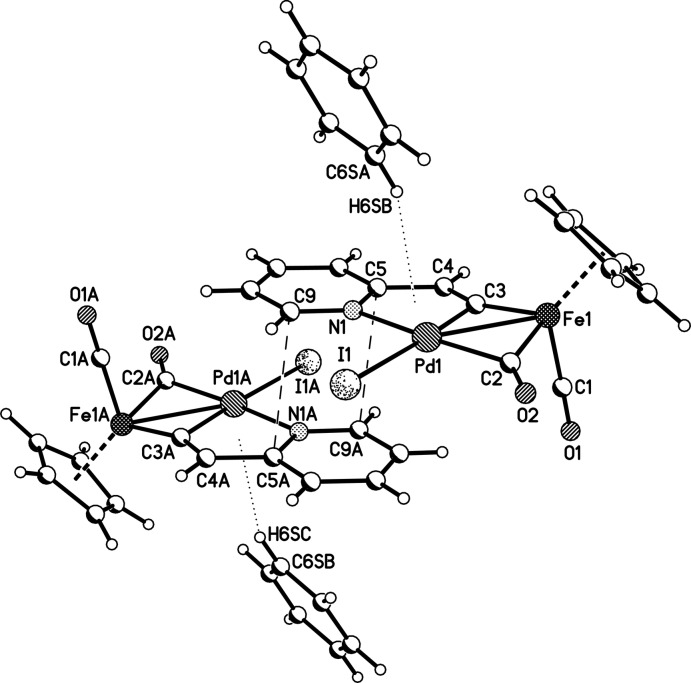
Centrosymmetric stacked dimer in the crystal packing. Atoms labelled with the suffix A are generated by the symmetry operation (1 − *x*, −*y*, −*z*).

In the crystal, the complexes form centrosymmetrical dimers (Fig. 3 ▸) due to π-stacking inter­actions between the pyridyl­vinyl­idene fragments with an inter­planar distance of 3.36 Å and a shortest inter­atomic C5⋯C9(1 − *x*, −*y*, −*z*) distance of 3.339 (4) Å. The outer plane of the pyridyl­vinyl­idene fragment in the dimer is additionally shielded by the solvating benzene mol­ecule, which is oriented by one of its C—H groups to the centroid a of the five-membered chelating palladacycle [the C6*S*—H6*SA*⋯*Cg*1 distance is 2.67 Å; *Cg*1 is the centroid of the five-membered ring, the angle between the *Cg*1⋯H6*SA* vector and the ring normal is 9.7°, and the C6*S*—H6*SA*⋯*Cg*1 angle is 160°].

## Synthesis and crystallization   

A mixture of Cp(CO)_2_FeI (127.3 mg, 0.419 mmol) and PdCl_2_ (76 mg, 0.429 mmol) in diisopropyl amine (4 ml) was heated to 315 K and H—C≡C(2-C_5_H_4_N) (0.3 ml) was added. The mixture was stirred for 16 h at 333 K and the diisopropyl amine was removed under reduced pressure. The crude mixture was extracted with di­chloro­methane, the extract was filtered through celite, and the solvent was evaporated to dryness. The residue was dissolved in a di­chloro­methane–hexane (1:1) mixture and chromatographed on a silica column (9.5 × 1 cm). A dark-yellow band was eluted with di­chloro­methane and the eluate was evaporated to yield Cp(CO)_2_Fe(μ-C=CH(2-C_5_H_4_N)PdI (**FePd1**) (29 mg, 12%) as a brown solid. Red–brown crystals of the complex suitable for X-ray diffraction analysis were obtained after recrystallization from a di­chloro­methane–benzene solvent mixture. IR (CH_2_Cl_2_, ν/cm^−1^): 2028*s*, 1880*s* (ν_CO_), 1600*m*, 1584*m*, 1548*m*, 1468*m* (ν_C=C_ and ν_C=N_).

## Refinement   

Crystal data, data collection and structure refinement details are summarized in Table 1 ▸. Atom H4 of the vinyl group was located in a difference Fourier map and refined freely. All other H atoms were fixed geometrically and refined using a riding model with *U*_iso_(H) = 1.2*U*_eq_(C).

## Supplementary Material

Crystal structure: contains datablock(s) I. DOI: 10.1107/S2056989016019915/hb7640sup1.cif

Structure factors: contains datablock(s) I. DOI: 10.1107/S2056989016019915/hb7640Isup2.hkl

CCDC reference: 1523136

Additional supporting information: crystallographic information; 3D view; checkCIF report

## Figures and Tables

**Table 1:** Experimental details

Crystal data	
Chemical formula	[FePd(C_5_H_5_)(C_7_H_5_N)I(CO)_2_]·C_6_H_6_
*M*_r_	591.49
Crystal system, space group	Monoclinic, *P*2_1_/*c*
Temperature (K)	100
*a*, *b*, *c* (Å)	14.3058 (8), 9.0983 (5), 14.7315 (8)
β (°)	100.553 (1)
*V* (Å^3^)	1885.00 (18)
*Z*	4
Radiation type	Mo *K*α
μ (mm^−1^)	3.38
Crystal size (mm)	0.24 × 0.18 × 0.08

Data collection	
Diffractometer	Bruker APEXII CCD
Absorption correction	Multi-scan (*SADABS*; Bruker, 2004 ▸)
*T*_min_, *T*_max_	0.578, 0.774
No. of measured, independent and observed [*I* > 2σ(*I*)] reflections	23041, 5501, 5075
*R*_int_	0.023
(sin θ/λ)_max_ (Å^−1^)	0.703

Refinement	
*R*[*F* ^2^ > 2σ(*F* ^2^)], *wR*(*F* ^2^), *S*	0.026, 0.066, 1.15
No. of reflections	5501
No. of parameters	239
H-atom treatment	H atoms treated by a mixture of independent and constrained refinement
Δρ_max_, Δρ_min_ (e Å^−3^)	1.74, −0.81

Computer programs: *APEX2* and *SAINT* (Bruker, 2004 ▸), *SHELXS97* and *SHELXTL* (Sheldrick, 2008 ▸) and *SHELXL2014* (Sheldrick, 2015 ▸).

## supplementary crystallographic information

### Crystal data

**Table:**

[FePd(C_5_H_5_)(C_7_H_5_N)I(CO)_2_]·C_6_H_6_	*F*(000) = 1136
*M**_r_* = 591.49	*D*_x_ = 2.084 Mg m^−^^3^
Monoclinic, *P*2_1_/*c*	Mo *K*α radiation, λ = 0.71073 Å
*a* = 14.3058 (8) Å	Cell parameters from 9930 reflections
*b* = 9.0983 (5) Å	θ = 2.6–33.2°
*c* = 14.7315 (8) Å	µ = 3.38 mm^−^^1^
β = 100.553 (1)°	*T* = 100 K
*V* = 1885.00 (18) Å^3^	Prism, red-brown
*Z* = 4	0.24 × 0.18 × 0.08 mm

### Data collection

**Table:**

Bruker APEXII CCD diffractometer	5075 reflections with *I* > 2σ(*I*)
φ and ω scans	*R*_int_ = 0.023
Absorption correction: multi-scan (SADABS; Bruker, 2004)	θ_max_ = 30.0°, θ_min_ = 1.5°
*T*_min_ = 0.578, *T*_max_ = 0.774	*h* = −20→20
23041 measured reflections	*k* = −12→12
5501 independent reflections	*l* = −20→20

### Refinement

**Table:**

Refinement on *F*^2^	0 restraints
Least-squares matrix: full	Hydrogen site location: mixed
*R*[*F*^2^ > 2σ(*F*^2^)] = 0.026	H atoms treated by a mixture of independent and constrained refinement
*wR*(*F*^2^) = 0.066	*w* = 1/[σ^2^(*F*_o_^2^) + (0.0294*P*)^2^ + 3.6027*P*] where *P* = (*F*_o_^2^ + 2*F*_c_^2^)/3
*S* = 1.15	(Δ/σ)_max_ < 0.001
5501 reflections	Δρ_max_ = 1.74 e Å^−^^3^
239 parameters	Δρ_min_ = −0.81 e Å^−^^3^

### Special details

**Table:**

Geometry. All esds (except the esd in the dihedral angle between two l.s. planes) are estimated using the full covariance matrix. The cell esds are taken into account individually in the estimation of esds in distances, angles and torsion angles; correlations between esds in cell parameters are only used when they are defined by crystal symmetry. An approximate (isotropic) treatment of cell esds is used for estimating esds involving l.s. planes.

### Fractional atomic coordinates and isotropic or equivalent isotropic displacement parameters (Å^2^)

**Table:**

	*x*	*y*	*z*	*U*_iso_*/*U*_eq_	
Pd1	0.30724 (2)	0.14951 (2)	0.06141 (2)	0.01181 (5)	
Fe1	0.25581 (3)	0.41750 (4)	0.02367 (3)	0.01291 (7)	
I1	0.29779 (2)	−0.05920 (2)	0.18779 (2)	0.01770 (5)	
O1	0.43529 (15)	0.5582 (2)	0.10253 (15)	0.0230 (4)	
O2	0.23406 (14)	0.3136 (2)	0.20723 (14)	0.0190 (4)	
N1	0.37283 (15)	0.0155 (2)	−0.03060 (15)	0.0140 (4)	
C1	0.36439 (19)	0.5038 (3)	0.07341 (18)	0.0162 (5)	
C2	0.25372 (17)	0.3042 (3)	0.13501 (18)	0.0149 (4)	
C3	0.32265 (18)	0.2919 (3)	−0.03820 (17)	0.0145 (4)	
C4	0.36562 (18)	0.2467 (3)	−0.10583 (17)	0.0154 (5)	
H4	0.376 (3)	0.311 (4)	−0.156 (3)	0.020 (9)*	
C5	0.39136 (18)	0.0925 (3)	−0.10481 (17)	0.0148 (4)	
C6	0.43071 (19)	0.0223 (3)	−0.17359 (18)	0.0178 (5)	
H6A	0.4448	0.0770	−0.2244	0.021*	
C7	0.44904 (19)	−0.1271 (3)	−0.16727 (19)	0.0196 (5)	
H7A	0.4746	−0.1763	−0.2141	0.024*	
C8	0.42948 (19)	−0.2043 (3)	−0.09119 (19)	0.0192 (5)	
H8A	0.4414	−0.3069	−0.0853	0.023*	
C9	0.39229 (19)	−0.1283 (3)	−0.02432 (19)	0.0169 (5)	
H9A	0.3801	−0.1805	0.0281	0.020*	
C10	0.11143 (19)	0.3898 (3)	−0.0416 (2)	0.0211 (5)	
H10A	0.0767	0.2940	−0.0511	0.025*	
C11	0.1632 (2)	0.4550 (3)	−0.1039 (2)	0.0213 (5)	
H11A	0.1706	0.4153	−0.1654	0.026*	
C12	0.2014 (2)	0.5884 (3)	−0.0635 (2)	0.0222 (6)	
H12A	0.2398	0.6603	−0.0925	0.027*	
C13	0.1717 (2)	0.6070 (3)	0.0232 (2)	0.0248 (6)	
H13A	0.1859	0.6933	0.0655	0.030*	
C14	0.1163 (2)	0.4836 (3)	0.0370 (2)	0.0230 (6)	
H14A	0.0853	0.4656	0.0915	0.028*	
C1S	−0.1486 (2)	0.1192 (3)	0.2145 (2)	0.0238 (6)	
H1SA	−0.2048	0.1147	0.2400	0.029*	
C2S	−0.0785 (2)	0.2217 (3)	0.2473 (2)	0.0237 (6)	
H2SA	−0.0870	0.2876	0.2951	0.028*	
C3S	0.0040 (2)	0.2276 (3)	0.2099 (2)	0.0245 (6)	
H3SA	0.0518	0.2980	0.2321	0.029*	
C4S	0.0165 (2)	0.1307 (4)	0.1402 (2)	0.0261 (6)	
H4SA	0.0733	0.1334	0.1155	0.031*	
C5S	−0.0544 (2)	0.0301 (4)	0.1070 (2)	0.0272 (6)	
H5SA	−0.0469	−0.0347	0.0583	0.033*	
C6S	−0.1363 (2)	0.0238 (3)	0.1447 (2)	0.0242 (6)	
H6SA	−0.1842	−0.0464	0.1224	0.029*	

### Atomic displacement parameters (Å^2^)

**Table:**

	*U*^11^	*U*^22^	*U*^33^	*U*^12^	*U*^13^	*U*^23^
Pd1	0.01461 (9)	0.00833 (8)	0.01273 (9)	0.00015 (6)	0.00316 (6)	0.00055 (6)
Fe1	0.01464 (16)	0.00873 (15)	0.01475 (16)	0.00053 (12)	0.00109 (13)	0.00039 (12)
I1	0.02540 (9)	0.01143 (8)	0.01803 (9)	−0.00009 (6)	0.00861 (6)	0.00256 (6)
O1	0.0235 (10)	0.0192 (10)	0.0246 (10)	−0.0048 (8)	−0.0001 (8)	0.0015 (8)
O2	0.0224 (9)	0.0154 (9)	0.0191 (9)	0.0037 (7)	0.0035 (7)	−0.0003 (7)
N1	0.0162 (9)	0.0120 (9)	0.0140 (9)	0.0002 (8)	0.0033 (7)	−0.0005 (7)
C1	0.0212 (12)	0.0108 (11)	0.0165 (11)	0.0006 (9)	0.0033 (9)	0.0024 (9)
C2	0.0140 (10)	0.0106 (10)	0.0195 (11)	−0.0002 (8)	0.0014 (9)	−0.0010 (9)
C3	0.0162 (11)	0.0103 (10)	0.0161 (11)	−0.0004 (8)	0.0004 (9)	0.0027 (9)
C4	0.0182 (11)	0.0147 (11)	0.0133 (11)	−0.0015 (9)	0.0030 (9)	0.0025 (9)
C5	0.0152 (11)	0.0157 (11)	0.0133 (11)	−0.0021 (9)	0.0019 (9)	−0.0002 (9)
C6	0.0191 (11)	0.0185 (12)	0.0160 (11)	−0.0005 (10)	0.0038 (9)	−0.0003 (9)
C7	0.0201 (12)	0.0192 (12)	0.0201 (12)	0.0020 (10)	0.0048 (10)	−0.0056 (10)
C8	0.0207 (12)	0.0141 (12)	0.0224 (13)	0.0023 (9)	0.0030 (10)	−0.0033 (10)
C9	0.0188 (11)	0.0126 (11)	0.0195 (12)	0.0000 (9)	0.0044 (9)	0.0003 (9)
C10	0.0171 (12)	0.0157 (12)	0.0280 (14)	−0.0008 (9)	−0.0029 (10)	0.0020 (10)
C11	0.0205 (12)	0.0232 (13)	0.0178 (12)	0.0037 (10)	−0.0027 (10)	0.0022 (10)
C12	0.0230 (13)	0.0136 (12)	0.0273 (14)	0.0019 (10)	−0.0027 (11)	0.0092 (10)
C13	0.0246 (13)	0.0135 (12)	0.0327 (15)	0.0083 (10)	−0.0042 (11)	−0.0032 (11)
C14	0.0177 (12)	0.0275 (14)	0.0233 (13)	0.0080 (11)	0.0026 (10)	0.0002 (11)
C1S	0.0204 (13)	0.0207 (13)	0.0311 (15)	0.0027 (10)	0.0065 (11)	0.0073 (11)
C2S	0.0275 (14)	0.0209 (13)	0.0229 (13)	0.0016 (11)	0.0046 (11)	0.0003 (11)
C3S	0.0231 (13)	0.0216 (13)	0.0278 (14)	−0.0055 (11)	0.0023 (11)	−0.0022 (11)
C4S	0.0245 (14)	0.0274 (15)	0.0283 (15)	−0.0056 (11)	0.0100 (11)	−0.0025 (12)
C5S	0.0352 (16)	0.0223 (14)	0.0255 (14)	−0.0081 (12)	0.0094 (12)	−0.0041 (11)
C6S	0.0227 (13)	0.0206 (13)	0.0280 (14)	−0.0041 (11)	0.0008 (11)	0.0045 (11)

### Geometric parameters (Å, º)

**Table:**

Pd1—C3	1.999 (2)	C8—C9	1.387 (4)
Pd1—C2	2.012 (3)	C8—H8A	0.9500
Pd1—N1	2.161 (2)	C9—H9A	0.9500
Pd1—Fe1	2.5779 (4)	C10—C11	1.411 (4)
Pd1—I1	2.6800 (3)	C10—C14	1.430 (4)
Fe1—C1	1.775 (3)	C10—H10A	1.0000
Fe1—C3	1.836 (3)	C11—C12	1.416 (4)
Fe1—C2	1.942 (3)	C11—H11A	1.0000
Fe1—C12	2.075 (3)	C12—C13	1.428 (5)
Fe1—C13	2.102 (3)	C12—H12A	1.0000
Fe1—C11	2.119 (3)	C13—C14	1.410 (4)
Fe1—C14	2.128 (3)	C13—H13A	1.0000
Fe1—C10	2.128 (3)	C14—H14A	1.0000
O1—C1	1.140 (3)	C1S—C6S	1.381 (5)
O2—C2	1.152 (3)	C1S—C2S	1.389 (4)
N1—C9	1.337 (3)	C1S—H1SA	0.9500
N1—C5	1.365 (3)	C2S—C3S	1.392 (4)
C3—C4	1.328 (4)	C2S—H2SA	0.9500
C4—C5	1.449 (4)	C3S—C4S	1.389 (4)
C4—H4	0.97 (4)	C3S—H3SA	0.9500
C5—C6	1.400 (4)	C4S—C5S	1.388 (4)
C6—C7	1.384 (4)	C4S—H4SA	0.9500
C6—H6A	0.9500	C5S—C6S	1.386 (4)
C7—C8	1.394 (4)	C5S—H5SA	0.9500
C7—H7A	0.9500	C6S—H6SA	0.9500
			
C3—Pd1—C2	92.66 (10)	C6—C5—C4	124.7 (2)
C3—Pd1—N1	77.66 (9)	C7—C6—C5	119.7 (3)
C2—Pd1—N1	169.95 (9)	C7—C6—H6A	120.1
C3—Pd1—Fe1	45.13 (7)	C5—C6—H6A	120.1
C2—Pd1—Fe1	48.15 (7)	C6—C7—C8	119.0 (2)
N1—Pd1—Fe1	122.54 (6)	C6—C7—H7A	120.5
C3—Pd1—I1	174.36 (7)	C8—C7—H7A	120.5
C2—Pd1—I1	92.79 (7)	C9—C8—C7	118.6 (2)
N1—Pd1—I1	96.83 (6)	C9—C8—H8A	120.7
Fe1—Pd1—I1	140.469 (12)	C7—C8—H8A	120.7
C1—Fe1—C3	89.11 (12)	N1—C9—C8	122.7 (2)
C1—Fe1—C2	91.94 (11)	N1—C9—H9A	118.6
C3—Fe1—C2	100.29 (11)	C8—C9—H9A	118.6
C1—Fe1—C12	97.17 (12)	C11—C10—C14	108.8 (3)
C3—Fe1—C12	109.26 (12)	C11—C10—Fe1	70.25 (15)
C2—Fe1—C12	149.12 (12)	C14—C10—Fe1	70.36 (15)
C1—Fe1—C13	95.50 (12)	C11—C10—H10A	125.6
C3—Fe1—C13	149.20 (12)	C14—C10—H10A	125.6
C2—Fe1—C13	109.93 (12)	Fe1—C10—H10A	125.6
C12—Fe1—C13	39.97 (13)	C10—C11—C12	107.2 (3)
C1—Fe1—C11	130.76 (12)	C10—C11—Fe1	70.95 (16)
C3—Fe1—C11	87.59 (11)	C12—C11—Fe1	68.61 (15)
C2—Fe1—C11	136.91 (11)	C10—C11—H11A	126.4
C12—Fe1—C11	39.46 (11)	C12—C11—H11A	126.4
C13—Fe1—C11	66.44 (12)	Fe1—C11—H11A	126.4
C1—Fe1—C14	126.99 (12)	C11—C12—C13	108.8 (3)
C3—Fe1—C14	143.55 (12)	C11—C12—Fe1	71.93 (16)
C2—Fe1—C14	84.98 (11)	C13—C12—Fe1	71.03 (16)
C12—Fe1—C14	66.00 (12)	C11—C12—H12A	125.6
C13—Fe1—C14	38.94 (12)	C13—C12—H12A	125.6
C11—Fe1—C14	65.88 (11)	Fe1—C12—H12A	125.6
C1—Fe1—C10	160.54 (11)	C14—C13—C12	107.5 (3)
C3—Fe1—C10	104.63 (11)	C14—C13—Fe1	71.51 (16)
C2—Fe1—C10	98.91 (11)	C12—C13—Fe1	68.99 (16)
C12—Fe1—C10	65.54 (11)	C14—C13—H13A	126.2
C13—Fe1—C10	65.65 (11)	C12—C13—H13A	126.2
C11—Fe1—C10	38.80 (11)	Fe1—C13—H13A	126.2
C14—Fe1—C10	39.26 (11)	C13—C14—C10	107.7 (3)
C1—Fe1—Pd1	97.81 (8)	C13—C14—Fe1	69.55 (16)
C3—Fe1—Pd1	50.52 (8)	C10—C14—Fe1	70.37 (16)
C2—Fe1—Pd1	50.48 (7)	C13—C14—H14A	126.1
C12—Fe1—Pd1	154.42 (9)	C10—C14—H14A	126.1
C13—Fe1—Pd1	156.51 (10)	Fe1—C14—H14A	126.1
C11—Fe1—Pd1	116.79 (8)	C6S—C1S—C2S	120.0 (3)
C14—Fe1—Pd1	118.88 (9)	C6S—C1S—H1SA	120.0
C10—Fe1—Pd1	101.57 (8)	C2S—C1S—H1SA	120.0
C9—N1—C5	119.3 (2)	C1S—C2S—C3S	119.9 (3)
C9—N1—Pd1	128.13 (18)	C1S—C2S—H2SA	120.1
C5—N1—Pd1	112.53 (17)	C3S—C2S—H2SA	120.1
O1—C1—Fe1	177.6 (3)	C4S—C3S—C2S	120.1 (3)
O2—C2—Fe1	141.7 (2)	C4S—C3S—H3SA	120.0
O2—C2—Pd1	137.0 (2)	C2S—C3S—H3SA	120.0
Fe1—C2—Pd1	81.37 (10)	C5S—C4S—C3S	119.6 (3)
C4—C3—Fe1	156.9 (2)	C5S—C4S—H4SA	120.2
C4—C3—Pd1	118.58 (19)	C3S—C4S—H4SA	120.2
Fe1—C3—Pd1	84.35 (10)	C6S—C5S—C4S	120.2 (3)
C3—C4—C5	116.3 (2)	C6S—C5S—H5SA	119.9
C3—C4—H4	122 (2)	C4S—C5S—H5SA	119.9
C5—C4—H4	121 (2)	C1S—C6S—C5S	120.2 (3)
N1—C5—C6	120.5 (2)	C1S—C6S—H6SA	119.9
N1—C5—C4	114.8 (2)	C5S—C6S—H6SA	119.9
